# Monitoring Influenza C and D Viruses in Patients With Respiratory Diseases in Japan, January 2018 to March 2023

**DOI:** 10.1111/irv.13345

**Published:** 2024-06-24

**Authors:** Kohei Shimizu, Chiharu Kawakami, Yoko Matsuzaki, Seiichiro Fujisaki, Shiho Nagata, Hiroko Morita, Kayo Watanabe, Hideka Miura, Tomoko Momoki, Miwako Saikusa, Hiroki Ozawa, Makoto Kumazaki, Shuzo Usuku, Nobuko Tanaka, Ryuichi Senda, Ichiro Okubo, Shinji Watanabe, Hideki Hasegawa, Yoshihiro Kawaoka, Emi Takashita

**Affiliations:** ^1^ Yokohama City Institute of Public Health Yokohama Kanagawa Japan; ^2^ Pandemic Preparedness, Infection, and Advanced Research Center The University of Tokyo Tokyo Japan; ^3^ Research Center for Global Viral Diseases National Center for Global Health and Medicine Research Institute Tokyo Japan; ^4^ Department of Infectious Diseases Yamagata University Faculty of Medicine Yamagata Japan; ^5^ Research Center for Influenza and Respiratory Viruses National Institute of Infectious Diseases Tokyo Japan; ^6^ Influenza Research Institute, Department of Pathobiological Sciences, School of Veterinary Medicine University of Wisconsin‐Madison Madison Wisconsin USA; ^7^ Division of Virology, Institute of Medical Science The University of Tokyo Tokyo Japan

**Keywords:** influenza C, influenza D, surveillance, zoonotic infection

## Abstract

**Background:**

Influenza viruses can cause zoonotic infections that pose public health risks. Surveillance of influenza A and B viruses is conducted globally; however, information on influenza C and D viruses is limited. Longitudinal monitoring of influenza C virus in humans has been conducted in several countries, but there has been no long‐term monitoring of influenza D virus in humans. The public health risks associated with the influenza D virus therefore remain unknown.

**Methods:**

We established a duplex real‐time RT‐PCR to detect influenza C and D viruses and analyzed respiratory specimens collected from 2144 patients in Japan with respiratory diseases between January 2018 and March 2023. We isolated viruses and conducted hemagglutination inhibition tests to examine antigenicity and focus reduction assays to determine susceptibility to the cap‐dependent endonuclease inhibitor baloxavir marboxil.

**Results:**

We detected three influenza C viruses belonging to the C/Kanagawa‐ or C/Sao Paulo‐lineages, which recently circulated globally. None of the specimens was positive for the influenza D virus. The C/Yokohama/1/2022 strain, isolated from the specimen with the highest viral RNA load and belonging to the C/Kanagawa‐lineage, showed similar antigenicity to the reference C/Kanagawa‐lineage strain and was susceptible to baloxavir.

**Conclusions:**

Our duplex real‐time RT‐PCR is useful for the simultaneous detection of influenza C and D viruses from the same specimen. Adding the influenza D virus to the monitoring of the influenza C virus would help in assessing the public health risks posed by this virus.

## 1 Introduction

The influenza C virus, first isolated from humans in 1947, causes local epidemics [1]. Influenza C virus primarily infects humans and causes relatively mild respiratory illness; however, it can cause bronchitis and pneumonia, particularly in children younger than 2 years or patients with underlying illnesses [2, 3]. Influenza D virus was first detected in swine in 2011 [4]. Although the primary host of the influenza D virus is bovines, it can cause zoonotic infections [4]. Influenza C and D viruses are genetically more closely related to each other than to influenza A or B viruses [5].

Longitudinal monitoring of the influenza C virus has been conducted in Australia [6], Austria [7], Hong Kong, China [8, 9], India [10], Japan [3, 11], the Philippines [12], Spain [13], and the USA [14]. Of the six lineages of influenza C viruses (C/Taylor, C/Mississippi, C/Aichi, C/Yamagata, C/Kanagawa, and C/Sao Paulo), the C/Kanagawa‐ and C/Sao Paulo‐lineage viruses recently cocirculated globally [3, 6, 7, 9, 10, 12]. In contrast, there has been no long‐term monitoring of the influenza D virus in humans.

High seroprevalence (94%–97%) of antibodies to influenza D virus was detected among cattle workers in the USA in 2011 [15]. A more comprehensive seroprevalence study in Italy between 2005 and 2017 revealed an increase in the seroprevalence of antibodies to the influenza D virus in the Italian population from 5.1% in 2005 to 46.0% in 2014 and identified a correlation between influenza D virus prevalence peaks in humans and epidemics in domestic pigs in Italy [16]. Moreover, influenza D virus genomes were detected in one (2.3%) of 44 bioaerosol samples from a hospital emergency room in the USA [17], one (4.2%) of 24 bioaerosol samples from an airport in the USA [18], four (14.3%) of 28 bioaerosol samples from poultry farms in Malaysia [19], seven (22.6%) of 31 bioaerosol samples from cattle workers in the USA [20], one (1.3%) of 78 nasal washes from pig farm workers in Malaysia [21], and 21 (67.7%) of 31 nasal washes from cattle workers in the USA [20]. These studies suggest that the influenza D virus may pose risks to public health.

Despite the potential public health risks of the influenza D virus, most studies have focused on animals. Only one retrospective screening for influenza C and D viruses has been reported; 3300 human respiratory specimens collected from hospitals and primary care facilities in southeast Scotland between August 2006 and June 2008 were analyzed using conventional RT‐PCR [22]. This study found influenza C virus genomes in six (0.2%) of the specimens, but no influenza D virus genomes were detected. To date, little is known about influenza D virus infections in humans.

Here, we established a duplex real‐time RT‐PCR to detect influenza C and D viruses and analyzed respiratory specimens from patients with respiratory diseases in Japan between January 2018 and March 2023. We isolated viruses and examined their antigenicity and antiviral susceptibility.

## 2 Methods

### 2.1 Clinical Specimens

Respiratory specimens (nasal swab, throat swab, nasal discharge, saliva, tracheal aspiration fluid, or sputum) were collected from 2144 patients with respiratory diseases in Yokohama, Japan, from January 2018 to March 2023 as part of the National Epidemiological Surveillance of Infectious Diseases and the Active Epidemiological Investigation for COVID‐19 in Japan. Yokohama is the largest municipality in Japan by population, with about 3.8 million residents and about 4700 agricultural workers. It is one of the largest international trade ports in Japan and has the largest Chinatown in East Asia, attracting about 30 million domestic and international tourists each year. Specimens were negative for influenza A and B viruses and severe acute respiratory syndrome coronavirus 2 (SARS‐CoV‐2).

### 2.2 Duplex Real‐Time RT‐PCR

Influenza C and D viruses were detected by using a duplex real‐time RT‐PCR. Total nucleic acids were extracted from 150 μL of clinical specimens by using MagDEA Dx SV and magLEAD (Precision System Science, Japan) and eluted to 50 μL. The nucleoprotein (NP) gene of the influenza C virus and the polymerase basic protein 1 (PB1) gene of influenza D virus were amplified from the extracted RNA by using a One Step PrimeScript III RT‐qPCR Mix (Takara Bio, Japan) in 20‐μL reactions containing 10 μL of 2 × One Step PrimeScript III RT‐qPCR Mix, 2.8 μL of primers and probe mixture for each influenza C and D virus (1.2 μL of 10 μM forward primer, 1.2 μL of 10 μM reverse primer, and 0.4 μL of 5 μM probe), 3.4 μL of nuclease‐free water, and 1 μL of RNA. The primers and probe for influenza C virus (FluCNP+1068, FluCNP‐1161, and FluCNP1100probe) were previously described [23], and those for influenza D virus (PB1‐Fwd: 5′‐CAGCTGCRATGTCYGTCATAAG‐3′, PB1‐Rev: 5′‐ACAAATTCGCAGGGCCATTA‐3′, and PB1‐Probe: 5′‐HEX‐AATGGACTTTCTCCTGGGACTGCT‐TAMRA‐3′) were modified in this study to detect viruses in all lineages [24]. The reaction was performed using a LightCycler 480 System II (Roche Diagnostics, Germany) and the following conditions: 5 min of reverse transcription at 52 °C, 10 s of denaturation at 95 °C, and 40 cycles at 95 °C for 5 s and 60 °C for 30 s. For positive control RNAs of influenza C and D viruses, the complete NP gene of C/Yamagata/15/2004 and the complete PB1 gene of D/bovine/Yamagata/1/2019 were synthesized by in vitro transcription using a T7 RiboMAX Express Large Scale RNA Production System (Promega, USA). The isolate ID in the GISAID EpiFlu Database (https://gisaid.org) for C/Yamagata/15/2004 is EPI_ISL_65156, and the accession numbers in GenBank for D/bovine/Yamagata/1/2019 are LC494105 and LC494111. The detection limits for the RNA genomes of the six lineages of influenza C viruses (C/Taylor, C/Mississippi, C/Aichi, C/Yamagata, C/Kanagawa, and C/Sao Paulo) and the four lineages of influenza D viruses (D/OK, D/660, D/Yama2016, and D/Yama2019) are shown in Table 1. D/swine/Oklahoma/1334/2011 and D/bovine/Nebraska/9‐5/2012 were kindly provided by Dr. Benjamin Hause (South Dakota State University, USA), and D/bovine/Yamagata/10710/2016 and D/bovine/Yamagata/1/2019 were kindly provided by Drs. Taisuke Horimoto and Shin Murakami (The University of Tokyo, Japan). Specimens positive for influenza C or D virus genomes were then subjected to quantitative real‐time RT‐PCR to determine the viral RNA load and real‐time RT‐PCR using FTD Respiratory Pathogens 21 (Fast Track Diagnostics, Malta) to confirm codetection with other respiratory viruses (coxsackievirus A and B; echovirus; enterovirus; human coronavirus 229E, HKU1, NL63, and OC43; human metapneumovirus; human parainfluenza virus 1, 2, 3, and 4; human parechovirus; human respiratory syncytial virus; rhinovirus; human adenovirus; and human bocavirus).

**TABLE 1:** Detection limits of the duplex real‐time RT‐PCR assay for the influenza C and D RNA genomes of each lineage.

Type	Lineage	Virus strain	Detection limits (copies/μL reaction)*
C	C/Taylor	C/Ann Arbor/1/50	11.70 ± 0.07
	C/Yamagata	C/Yamagata/15/2004	11.88 ± 0.07
	C/Sao Paulo	C/Yamagata/32/2014	11.88 ± 0.09
	C/Aichi	C/Yamagata/4/92	11.58 ± 0.05
	C/Kanagawa	C/Yamagata/13/2014	11.82 ± 0.02
	C/Mississippi	C/Yamagata/3/2000	11.89 ± 0.08
D	D/OK	D/swine/Oklahoma/1334/2011	12.10 ± 0.06
	D/660	D/bovine/Nebraska/9‐5/2012	12.17 ± 0.11
	D/Yama2016	D/bovine/Yamagata/10710/2016	11.97 ± 0.09
	D/Yama2019	D/bovine/Yamagata/1/2019	11.99 ± 0.19

* Mean ± SD copy numbers of triplicate reactions were determined.

### 2.3 Quantitative Real‐Time RT‐PCR

Quantitative real‐time RT‐PCR was established to determine the viral RNA load in the clinical specimens. Briefly, the NP gene of influenza C virus and the PB1 gene of influenza D virus were amplified from the extracted RNA by using AgPath‐ID One‐Step RT‐PCR Reagents (Thermo Fisher Scientific, USA) in 10‐μL reactions containing 5 μL of 2 × RT‐PCR Buffer, 0.4 μL of 25 × RT‐PCR Enzyme Mix, 1.4 μL of primers and probe mixture (0.6 μL of 10 μM forward primer, 0.6 μL of 10 μM reverse primer, and 0.2 μL of 5 μM probe), 2.2 μL of nuclease‐free water, and 1 μL of RNA. The reaction was performed using a LightCycler 480 System II and the following conditions: 10 min of reverse transcription at 45 °C, 10 min of denaturation at 95 °C, and 45 cycles at 95 °C for 15 s and 60 °C for 45 s. The viral RNA load was determined based on a standard curve generated by serial 10‐fold dilutions of the positive control RNA. The results are expressed as viral RNA load per microliter of reaction.

### 2.4 Deep Sequencing Analysis

The cDNA was amplified from the extracted RNA using a SuperScript III One‐Step RT‐PCR System with Platinum Taq DNA Polymerase (Thermo Fisher Scientific) and specific primers for influenza C virus genomes [8]. The DNA libraries were prepared from the whole genome amplicons of influenza C viruses by using a QIAseq FX DNA Library Kit (Qiagen, Germany), followed by purification using an AMPure XP (Beckman Coulter, USA). The library was sequenced using a MiSeq Reagent Kit v2 with MiSeq or iSeq 100 System (Illumina, USA). Sequence reads were aligned according to the reference sequences of C/Kanagawa/1/76 or C/Sao Paulo/378/82 using the QIAGEN CLC Genomics Workbench (Qiagen). The isolate IDs in the GISAID EpiFlu Database for C/Kanagawa/1/76 and C/Sao Paulo/378/82 are EPI_ISL_66328 and EPI_ISL_66344, respectively.

### 2.5 Hemagglutination Inhibition (HI) Test

To analyze the antigenicity of the influenza C virus, an HI test was performed in 96‐well microtiter plates using 0.5% chicken erythrocytes as previously described [25]. C/Kanagawa/1/76 and C/Sao Paulo/378/82 served as reference C/Kanagawa‐ and C/Sao Paulo‐lineage viruses, respectively [11]. Reference antisera against C/Kanagawa/1/76 and C/Sao Paulo/378/82 [11] or anti–hemagglutinin‐esterase‐fusion (HEF) monoclonal antibodies (J14, J9, Q5, U4, U9, U1, U2, MS22, YA3, and YA5) [1, 25] were used.

### 2.6 Focus Reduction Assay

The antiviral susceptibility of the influenza C virus was determined by using a focus reduction assay as previously described [26]. Briefly, confluent monolayers of swine testicular (ST) cells [4] in 96‐well plates were infected with 1000 focus‐forming units of virus/well. Virus adsorption was carried out for 1 h at 37 °C, and then an equal volume of Avicel RC‐581 (DuPont Nutrition USA, USA) in a culture medium containing serial dilutions of baloxavir acid (0.025–2500 nM) supplemented with 0.5 μg/mL TPCK‐trypsin was added to each well in triplicate. The cells were incubated for 18 h at 34 °C and then immunostained with the anti‐NP monoclonal antibody H27, followed by a horseradish peroxidase‐labelled goat anti‐mouse immunoglobulin (SeraCare Life Sciences, USA). The infected cells were stained with TrueBlue Substrate (SeraCare Life Sciences), and the focus numbers were quantified by using an ImmunoSpot S6 Analyzer, ImmunoCapture software, and BioSpot software (Cellular Technology, USA). The results are expressed as 50% inhibitory concentration (IC_50_) values, which were calculated by using GraphPad Prism (GraphPad Software, USA). C/Yamagata/13/2014 and C/Yamagata/32/2014 served as references for the C/Kanagawa‐ and C/Sao Paulo‐lineage viruses, respectively [26].

## 3 Results

### 3.1 Detection of Influenza C and D Virus Genomes in Clinical Specimens

To detect influenza C and D viruses in humans, we analyzed respiratory specimens collected from 2144 patients in Yokohama, Japan, who presented with respiratory diseases between January 2018 and March 2023 by using duplex real‐time RT‐PCR. The characteristics of the patients with respiratory diseases are shown in Table 2.

**TABLE 2:** Characteristics of patients with respiratory diseases between January 2018 and March 2023 in this study.

Characteristic	No. of patients	%
Sex		
Male	1120	52.2
Female	1024	47.8
Age group (years)		
0–1	386	18.0
2–5	356	16.6
6–19	293	13.7
20–64	712	33.2
≥ 65	395	18.4
Unknown	2	0.1

We detected influenza C virus genomes in three (0.1%) of the 2144 specimens: one each in 2020, 2022, and 2023 (Table 3). None of the specimens were positive for influenza D virus genomes. The frequency of influenza C virus was one (0.1%) of 1028, one (0.4%) of 250, and one (2.6%) of 38 in 2020, 2022, and 2023, respectively. Based on the HEF gene sequences, these viruses were classified into the C/Kanagawa‐ or C/Sao Paulo‐lineages, which were recently cocirculated globally (Table 3).

**TABLE 3:** Influenza C viruses detected in Yokohama, Japan, from January 2018 to March 2023.

GISAID isolate ID	Virus	Onset of symptoms	Specimen collection date	Sex	Age (years)	Lineage	Viral RNA load (copies/μL reaction)
EPI_ISL_18454398	C/Yokohama/1/2020	March 15, 2020	March 20, 2020	M	47	C/Sao Paulo	3.0 × 10^2^
EPI_ISL_18454397	C/Yokohama/1/2022	December 22, 2022	December 23, 2022	M	4	C/Kanagawa	2.9 × 10^4^
EPI_ISL_18454399	C/Yokohama/1/2023	February 7, 2023	February 9, 2023	M	6	C/Kanagawa	2.4 × 10^3^

Abbreviation: GISAID, Global Initiative on Sharing All Influenza Data.

### 3.2 Clinical Condition of the Patients Infected With Influenza C Viruses

C/Yokohama/1/2020 and C/Yokohama/1/2022 were detected in sporadic cases. C/Yokohama/1/2020 was detected in a 47‐year‐old male without comorbidities who developed pneumonia with a fever of 38.5 °C. SARS‐CoV‐2 infection was suspected, but C/Yokohama/1/2020 was detected. The patient infected with C/Yokohama/1/2022, a 4‐year‐old male without comorbidities, developed bronchitis with a fever of 38.0 °C. C/Yokohama/1/2023 was detected in a 6‐year‐old male with bronchial asthma. He developed an upper respiratory illness with a fever of 39.0 °C. His sister exhibited similar upper respiratory symptoms, but we could not obtain a specimen from her. No other respiratory viruses (coxsackievirus A and B; echovirus; enterovirus; human coronavirus 229E, HKU1, NL63, and OC43; human metapneumovirus; human parainfluenza virus 1, 2, 3, and 4; human parechovirus; human respiratory syncytial virus; rhinovirus; human adenovirus; and human bocavirus) were detected with C/Yokohama/1/2020 and C/Yokohama/1/2022; however, human coronavirus NL63 was detected with C/Yokohama/1/2023.

### 3.3 Isolation and Characterization of an Influenza C Virus Strain

We attempted to isolate influenza C virus strains from the clinical specimens that were positive for influenza C virus genomes by inoculation into the amniotic cavity of 8‐day‐old embryonated hen's eggs at 34 °C, because isolation and passage of influenza C virus in eggs can yield viruses with high hemagglutination titers without changing their antigenicity [11]. Of the three specimens positive for influenza C virus genomes, C/Yokohama/1/2022 was isolated from the specimen with the highest viral RNA load (Table 3). The C/Yokohama/1/2022 strain showed similar antigenicity to the reference strain C/Kanagawa/1/76, a representative C/Kanagawa‐lineage virus, when tested against the antibodies used (Table 4).

**TABLE 4:** Antigenicity of the influenza C virus strain isolated in Yokohama, Japan.

Virus strain	Lineage	HI titers using antisera against:		HI titers using anti‐HEF monoclonal antibodies									
		C/Kanagawa/1/76	C/Sao Paulo/378/82	A‐1*					A‐3*			Y‐1*	
				J14	J9	Q5	U4	U9	U1	U2	MS22	YA3	YA5
Reference viruses													
C/Kanagawa/1/76	C/Kanagawa	2560	320	128,000	< 20	320	20	40	< 20	< 20	< 20	< 20	< 20
C/Sao Paulo/378/82	C/Sao Paulo	1280	1280	64,000	< 20	800	3200	16,000	32,000	12,800	12,800	12,800	6400
Test virus													
C/Yokohama/1/2022	C/Kanagawa	2560	320	64,000	< 20	160	20	80	< 20	< 20	< 20	80	40

Abbreviations: HEF, hemagglutinin‐esterase‐fusion; HI, hemagglutination inhibition.

* Representative antigenic sites of HEF.

Previous studies suggest that the cap‐dependent endonuclease inhibitor baloxavir marboxil could be used for the treatment of influenza C and D virus infections [26, 27]. We therefore examined the baloxavir susceptibility of the C/Yokohama/1/2022 strain and the C/Kanagawa‐ and C/Sao Paulo‐lineage reference strains used in our previous study [26] (Table 5). The C/Yokohama/1/2022 strain showed comparable IC_50_ values to the reference strains.

**TABLE 5:** Baloxavir susceptibility of the influenza C virus strain isolated in Yokohama, Japan.

Virus strain	Lineage	Mean IC_50_ ± SD (nM)*
Reference viruses		
C/Yamagata/13/2014	C/Kanagawa	8.86 ± 1.27
C/Yamagata/32/2014	C/Sao Paulo	5.19 ± 0.71
Test virus		
C/Yokohama/1/2022	C/Kanagawa	7.68 ± 1.43

Abbreviations: IC_50_, 50% inhibitory concentration; SD, standard deviation.

* Mean IC_50_ values of triplicate reactions were determined by use of a focus reduction assay.

## 4 Discussion

Influenza viruses pose a risk to public health because they cause zoonotic infections. Although the World Health Organization Global Influenza Surveillance and Response System (GISRS) conducts global surveillance of influenza A and B viruses, information on influenza C and D viruses is limited. In particular, little is known about influenza D virus infections in humans. To address this knowledge gap, we monitored these viruses in patients with respiratory diseases in Japan between January 2018 and March 2023. We established a duplex real‐time RT‐PCR to detect influenza C and D viruses. The detection limits for the RNA genomes of influenza C and D viruses were 11.63–11.96 copies/μL and 12.05–12.19 copies/μL, respectively (Table 1). These results demonstrate the usefulness of our duplex real‐time RT‐PCR for the simultaneous detection of both viruses in the same specimen.

We found that the frequency of influenza C virus between January 2018 and March 2023 in Yokohama, Japan, was three (0.1%) of 2144 specimens. After the first case of SARS‐CoV‐2 infection was detected in February 2020, the frequency of influenza A and B viruses was appreciably reduced in Yokohama, Japan; however, the frequency of these viruses between January 2018 and December 2019 was 489 (36.7%) of 1331 respiratory specimens in this region [28]. In Australia between 2008 and 2014 [6], Austria between the 2016/2017 and 2021/2022 influenza seasons [7], Hong Kong between 2014 and 2019 [8], India between 2009 and 2015 [10], Spain between 2005 and 2012 [13], and the USA between 2006 and 2012 [14], the frequency of influenza C virus was 79 (0.6%) of 13,497, 108 (0.5%) of 22,565, 2394 (0.2%) of 1,088,090, 3 (0.1%) of 2530, 17 (0.6%) of 2687, and 13 (0.3%) of 4200 specimens, respectively.

We did not detect influenza D viruses in this study, which is consistent with a retrospective screening of 3300 human respiratory specimens collected between August 2006 and June 2008 in southeast Scotland [22]. Although global surveillance of influenza C and D viruses has not been conducted, these results indicate that the frequency of influenza C virus is lower than that of influenza A and B viruses and that the frequency of influenza D virus may be even lower than that of influenza C virus. Influenza D virus was first detected in swine, and its primary host is cattle [4]. Our study is geographically limited, and the exposure profiles of the patients from whom specimens were collected to these animals are unknown. Further studies are needed to detect the influenza D virus in human populations.

In Austria, influenza A and C viruses were not detected during the 2020/2021 season, and most cases diagnosed from sentinel samples were SARS‐CoV‐2; however, the frequency of influenza A and C viruses increased in the following 2021/2022 season [7]. The frequency of influenza C virus before the COVID‐19 pandemic was three (0.3%) of 1120, eight (0.4%) of 1952, four (0.1%) of 3278, and two (0.04%) of 4950 in the 2016/2017, 2017/2018, 2018/2019, and 2019/2020 seasons, respectively. In contrast, its frequency was 91 (2.2%) of 4077 in the 2021/2022 season [7]. These results suggest that the risk of influenza C virus infections increases as the number of SARS‐CoV‐2 cases decreases. In Japan, influenza activity was low throughout the COVID‐19 pandemic; however, during the 2022/2023 season, Japan experienced its first influenza outbreak since the 2019/2020 season. We detected one influenza C virus between 2018 and early 2020, but two viruses in the 2022/2023 season alone. Therefore, the frequency of influenza C or even D viruses may increase during the upcoming season. These findings highlight the significance of close monitoring of these viruses.

Influenza C virus is a significant cause of upper respiratory illness in children younger than 6 years, and the risk of complications with lower respiratory illness is particularly high in children younger than 2 years [2, 3]. In this study, we detected three influenza C viruses. The patients infected with C/Yokohama/1/2020 and C/Yokohama/1/2022 developed lower respiratory illnesses, whereas the patients infected with C/Yokohama/1/2023 developed upper respiratory illnesses. No additional respiratory viruses were detected in the specimens collected from the patients with lower respiratory illnesses. These findings indicate that C/Yokohama/1/2020 and C/Yokohama/1/2022 caused lower respiratory illnesses. A comparison of whole genome sequences among these three viruses would be helpful in understanding their pathogenicity; however, those of C/Yokohama/1/2020 and C/Yokohama/1/2023 could not be obtained due to their low viral RNA load (Table 3).

We previously reported the baloxavir susceptibilities of all six lineages of influenza C viruses and four lineages of influenza D viruses (D/OK, D/660, D/Yama2016, and D/Yama2019) [26]. No significant differences in the IC_50_ values for baloxavir were found among the distinct lineages of influenza C or D viruses. Furthermore, all influenza C and D viruses tested were susceptible to baloxavir. In this study, the C/Yokohama/1/2022 strain showed comparable IC_50_ values to the C/Kanagawa‐ and C/Sao Paulo‐lineage reference strains whose baloxavir susceptibilities were determined in our previous study [26] (Table 5). Our results indicate that C/Yokohama/1/2022 is susceptible to baloxavir. Clinical data on the effectiveness of this drug to treat patients infected with influenza C and D viruses should be obtained in the future.

Because different lineages of influenza C and D viruses cocirculate, reassortment within and between these lineages can occur and impact transmission [5]. Furthermore, influenza D virus prevalence peaks in humans and appears to follow enzootics in animals [16]. Increased outbreaks in animals raise concerns about influenza D virus adaptation in humans. Consequently, continued monitoring of the influenza D virus in both humans and animals is essential to evaluating the public health risks posed by this virus.

## 5 Conclusion

Our duplex real‐time RT‐PCR is useful for the simultaneous detection of influenza C and D viruses from the same specimen. Adding the influenza D virus to the monitoring of the influenza C virus would help assess the public health risks posed by this virus.

## Author Contributions

**Kohei Shimizu:** investigation, writing–review and editing. **Chiharu Kawakami:** investigation, writing–review and editing. **Yoko Matsuzaki:** investigation, writing–review and editing. **Seiichiro Fujisaki:** investigation, writing–review and editing. **Shiho Nagata:** investigation, writing–review and editing. **Hiroko Morita:** investigation, writing–review and editing. **Kayo Watanabe:** investigation, writing–review and editing. **Hideka Miura:** investigation, writing–review and editing. **Tomoko Momoki:** investigation, writing–review and editing. **Miwako Saikusa:** investigation, writing–review and editing. **Hiroki Ozawa:** investigation, writing–review and editing. **Makoto Kumazaki:** investigation, writing–review and editing. **Shuzo Usuku:** investigation, writing–review and editing. **Nobuko Tanaka:** writing–review and editing. **Ryuichi Senda:** writing–review and editing. **Ichiro Okubo:** writing–review and editing. **Shinji Watanabe:** writing–review and editing. **Hideki Hasegawa:** writing–review and editing. **Yoshihiro Kawaoka:** conceptualization, writing–review and editing. **Emi Takashita:** conceptualization, investigation, writing–original draft.

## Conflicts of Interest

The authors declare no conflicts of interest.

## Data Availability

Data is available on request from the authors.
